# School Dental Services Theoretical Model-Based on Geographic Information System in Al-Madinah, Saudi Arabia

**DOI:** 10.3390/children10020186

**Published:** 2023-01-19

**Authors:** Amal Aqeeli, Alla T. Alsharif, Marc Tennant, Estie Kruger

**Affiliations:** 1 International Research Collaborative—Oral Health and Equity, School of Human Sciences, The University of Western Australia, Perth, WA 6009, Australia; 2 Preventive Dental Sciences, Taibah University Dental College and Hospital, Al-Madinah Al-Munawwarah 42353, Saudi Arabia

**Keywords:** SDS, GIS, dental services, access, health system

## Abstract

The study aimed to design a geographic theoretical model for school dental services (SDS) in Al-Madinah, Saudi Arabia (SA), using a geographic information system (GIS). The location of all primary public schools and the student population at each school were obtained from the General Administration of Education in Al-Madinah Al-Munawwarah Region website. The geographic modeling for SDS was analyzed using GIS according to two models. A scenario was developed to simulate the demand for dental care for the two models based on schoolchildren’s estimated oral health profile. The areas with the higher number of schools; higher number of students; and dense child population as presented in the map suggest the future location of SDS. The total number of dentists required to work in SDS settings was 415 for the first model, and 277 for the second model. The suggested average number of dentists per district in the highest child population density districts is 18 dentists in the first model, compared to 14 in the second model. Establishing SDS is suggested as a solution to the persistently high prevalence of dental caries among schoolchildren in Al-Madinah and SA in general. A model was suggested for SDS with a guide of the proposed SDS locations and the number of dentists to hire for the services to meet the child population’s oral health needs.

## 1. Introduction

Public health is defined as an “organized response by society to protect and promote health, and to prevent injury, illness, and disability” [1]. It aims to provide the necessary environment for the population to be healthy [2]. Despite the significance of genal and dental public health, they still lack community and political support compared to other health sectors in terms of investments.

One of the most important public health outcomes is the protection and improvement of children’s health. The past few decades have shown great progress in children’s health including reducing morbidity and mortality rates. However, work remains to be done to further improve children’s health. Common health problems for schoolchildren around the world include respiratory and ear infections, gastrointestinal problems such as diarrhea and vomiting, and allergies with reactions that range from mild to severe, bacterial, viral, and parasite infections. In Saudi Arabia (SA), a National School-Based Screening Program was conducted by The Ministry of Health for health screening of schoolchildren’s general health [3]. The most common found health problems were dental caries, eye refractory errors, and overweightness and obesity [3].

Schoolchildren in SA have a very high prevalence of dental caries [4]. In Al-Madinah, caries prevalence among 9–12-year-old school children is 85%, and 1 in 4 children have never visited the dentist [5]. Current caries management methods in SA include school oral health promotion programs, fluoridated toothpaste, fluoridated water, and free dental care provision for Saudi citizens. However, the persistently high prevalence of dental caries among children in SA indicates that these methods are insufficient or ineffective [6].

Providing healthcare in schools has been a well-established method for delivering healthcare to children worldwide. School-based health centers in The United States (US) and school dental services (SDS) in Australia and other countries have been created as a model for delivering free healthcare to children [7,8]. Strong evidence suggests the benefits of these school-based centers, which include financial, physical health (including medical and dental), mental health, and educational outcomes [7].

Schools, where children spend most of their time, provide a firmly established infrastructure to support the provision of oral health-care. School oral health programs in some areas have fixed centers and mobile vans that provide screening, fissure sealants, and topical fluoride applications [9]. SDS has been operated in several countries around the world with different cultural and economic backgrounds [8,10]. Significant progress in reducing dental caries in school children is evident in Australia and is mainly attributed to SDS alongside exposure to fluoridated water [11,12].

In recent decades, a geographic information system (GIS) has been implemented in dental health planning as a computational tool to examine the spatial aspect of access to dental care services [13]. GIS was initially designed in the late 20th century and ever since it has been universally relevant, however, its application in the dental research field is considered fairly recent [14]. This study aimed to design a GIS-based theoretical model for SDS in Al-Madinah, SA.

## 2. Materials and Methods

This was a geographical theoretical model development study for SDS conducted in Al-Madinah, SA using GIS. Al-Madinah, also spelled Medina, is the fourth most populous city in SA and it is the main city of Al-Madinah Al-Munawwarah Region with a total population of 1,105,465, which represents 3.5% of the total Saudi population [15]. The urban catchment area of Al-Madinah covers 293 km and has a population growth rate of 2.3% over the last few years [15]. The city has a significant Islamic heritage being the second holiest city in Islam after Makkah. It is the home of the Prophet Mohammad’s Mosque (Al-Masjid An-Nabawi) which is located in the heart of the city with the city’s population circulating the mosque.

### 2.1. Model’s Data

*Census collection district*

Al-Madinah’s urban area was divided into 103 collection districts. Each district was allocated a unique identifier and the geographic boundaries of those districts were obtained from the General Authority of Statistics’ website [16].

*Population data*

The population data within the metropolitan area of Al-Madinah were obtained from the most recent (2010) census data available on the General Authority of Statistics’ website [15]. Because access to child population data could not be obtained at the city level, an estimation of the number of children aged between 0–17 years old was made based on the SA United Nations Children’s Fund country profile document [17]. The child population data were divided by census collection districts and defined by geographic boundaries.

*Primary schools data*

The location of all primary public schools and the student population at each school were obtained from the Ministry of Education, the General Administration of Education in Al-Madinah Al-Munawwarah Region website [18]. Geocoding of the schools’ locations was completed using a free-access, geocoding website for Google Maps, by which geographic coordinates (longitudes and latitudes) were assigned to the physical addresses of the locations.

*Mapping*

Spatial and non-spatial data were entered into Quantum-GIS (QGIS) software. The spatial data include the coordinates of the schools and the districts. The nonspecial data include population data, student population, and the two models’ data that will be explained in the next section. A shape file for Al-Madinah map with districts’ boundaries was added as a vector layer on QGI. The child population data for each district was added and randomly distributed across each district. The schools’ coordinates were entered in an Excel sheet, converted to a CSV file, and added as a delaminated layer with points coordinates on QGIS. An additional layer of student population size was added, shown as circles around each school.

### 2.2. SDS Geographic Modeling

The geographic theoretical modeling for SDS in Al-Madinah was built according to two models. A scenario was developed to simulate the demand for dental care for each model, based on the estimated oral health profile of schoolchildren.

*Model 1*

This model demonstrates meeting current oral health needs (based on the high caries profile for schoolchildren in Al-Madinah as found in previous studies [19,20]). The assumption that was considered in this model was that high levels of caries would lead to a high need and demand for dental care, requiring greater numbers of SDS and health workers. The number of dentists required for the model was expressed as full-time equivalent (FTE) and calculated as described in the equation:

Service days = total children dental visits per year/dental visits per child per year

(1)FTE = service days/working days

The patients that a dentist would treat per day was estimated to be 15 patients per day and the estimated number of dental visits per child per year was 4.5 visits (one standard visit every six months for check-ups adding to that an estimated 2.5 visits for treating current caries conditions). The working days per calendar year in SA were estimated to be 237 days. The calculation was run on Excel for FTS per district.

Although this model suggests shorter wait time for appointments availability and quick service delivery to meet the current needs, it is considered a more costly and less sustainable model assuming that a transition would be observed in SDS on a city level from a treatment focus to a prevention focus approach once an improvement in the caries profile of schoolchildren has been achieved.

*Model 2*

Due to limited resources and the high initial cost of establishing SDS, a second model that is more sustainable and easier to maintain following the anticipated decline in oral health needs once SDS are established was suggested. This model demonstrates a smaller size SDS with a smaller number of workers. The number of FTE health professionals was calculated based on the assumption of 3 visits per child per year instead of the 4.5 yearly visits suggested in model 1. We suggest that children’s oral health would improve after receiving dental care from the newly established SDS and that children would need two essential check-up visits per year (adding to that one additional visit for any extra potential treatment needed).

While model 2 is more cost-effective than model 1, as it has a less initial cost and is easier to maintain, it will require more initial waiting time for appointments availability once the SDS is established and the transition from a treatment focus to prevention focus approach on a city level was observed and the oral health of the child population in the city has improved.

*Integrated model development*

A separate data sheet for each model contacting the calculated FTE per each district was converted to a CSV file, added as a delimitated text layer to the project on QGIS, and joined with the shape file of Al-Madinah map using the unique identifier per district.

*Data analysis*

Data entry and tabulation were performed using Microsoft Excel for Mac software version 16.66.1. Geographic mapping was performed using QGIS version 3.10. QGIS is a free; open-source geographic information system software that supports geospatial data analysis, licensed under the GNU General Public License version 2. The mapping was performed at a high-precision resolution and analysis that allowed high-precision analysis of the service distribution to the population. The project coordinate reference system was WGS 84, and the authority ID was EPSG: 4326. The methods used in this study were consistent with previous studies conducted in the same research field [21].

*Ethical consideration*

All the data used in this study was obtained from open-access online sources. An ethical exemption was obtained from the Taibah University Research Ethics Committee in Al-Madinah, SA (TUCDREC/20I 70305/Bakeer), and a clearance from the University of Western Australia Ethics Committee was attained (RA/4/20/5467). The study was conducted following the principles of the World Medical Association of Helsinki and the study follows the guidelines for observational studies of the Strengthening the Reporting of Observational Studies in Epidemiology (STROBE) [22].

## 3. Results

### 3.1. Population Data

The total number of public primary schools that were geocoded in Al-Madinah was 308. The total child population in the 0–17-year-old group in 2010 was estimated to be 330,206 in Al-Madinah. The city center, with an average of 15,500 children, had the highest child population density and the greatest number of primary schools.

### 3.2. Distribution of Primary Schools

**Figure 1:**
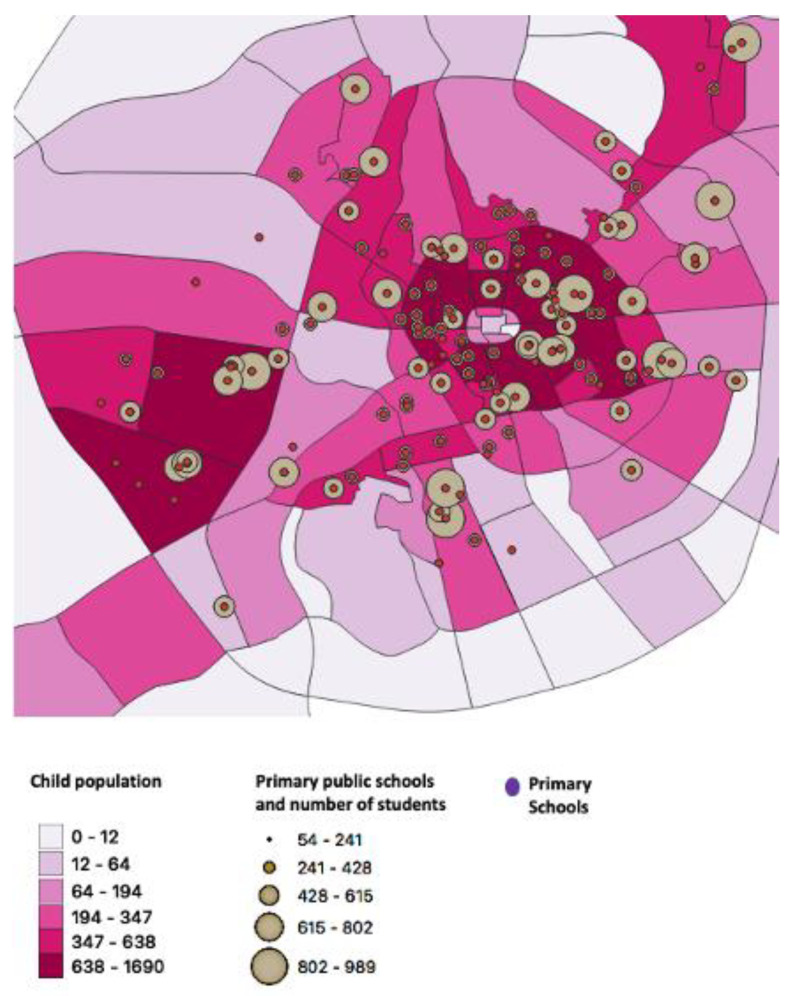
The distribution of the primary schools with the student population and the child population in Al-Madinah city.

The distribution of the primary schools with the student population and the child population in Al-Madinah city is presented in Figure 1. The size of the circle reflects the student population size at each respective school. The child population density per district is presented as a gradient, the higher density, the higher the child population size per district. The child population per district ranges from 16,902—5 children per district and the student population per school ranges from 989—54 students per school.

### 3.3. The Proposed SDS Location

The majority of children in Al-Madinah reside in and around the city center with a high population density. The areas with the higher number of schools, higher number of students, and dense child population as presented in the map, suggesting the future location of SDS.

### 3.4. SDS Geographic Modeling

*Model 1*

**Figure 2:**
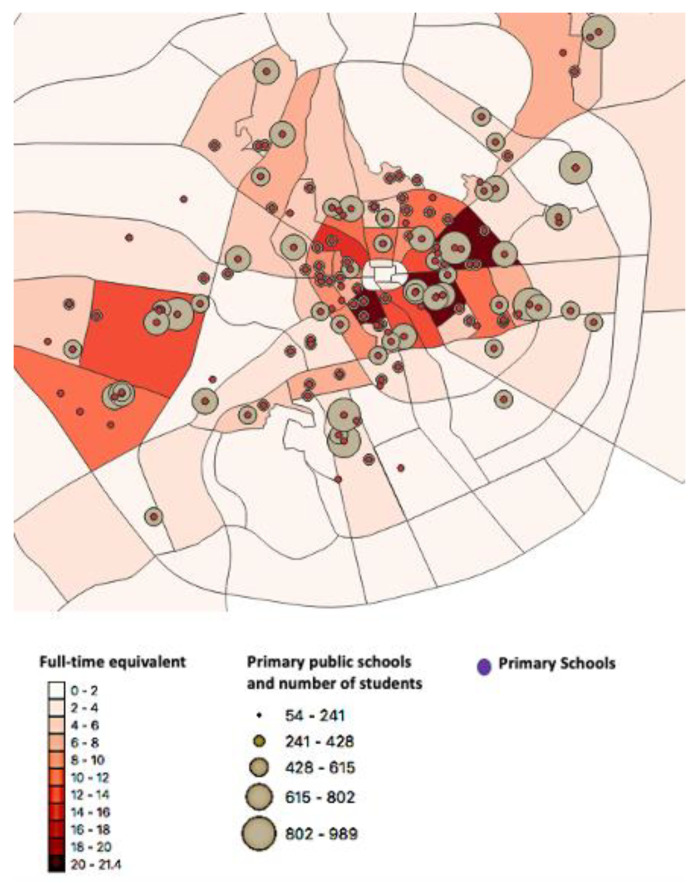
Model 1: a geographic model for school dental services (SDS) in Al-Madinah city presenting the distribution of full-time equivalent for SDS for each district.

Figure 2 shows the map for model 1. The total number of dental visits by children in Al-Madinah per year was estimated to be 1,478,709 (Table 1). The total number of dentists suggested to operate the SDS in Al-Madinah for this model was 415. The number of dentists suggested per district ranges from 21 for the high-density child population to 2 dentists per district. The top 10 districts with the highest child population require a suggested average number of 18 working dentists per district.

*Model 2*

**Figure 3:**
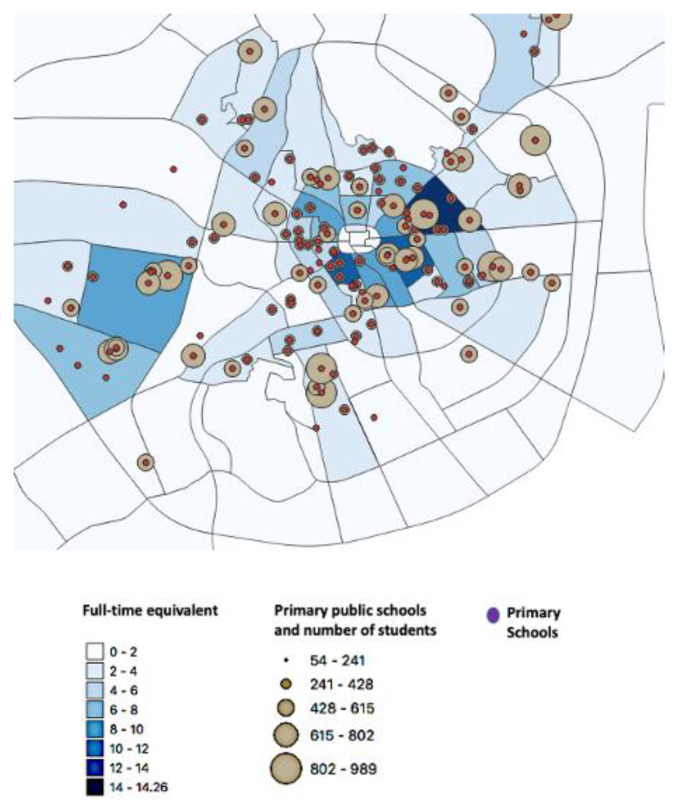
Model 2: a geographic model for school dental services (SDS) in Al-Madinah city presenting the distribution of full-time equivalent for SDS for each district.

Figure 3 shows the map for model 2. The total number of dental visits of children in Al-Madinah per year was estimated to be 985,806. The total number of dentists suggested to work at SDS in Al-Madinah was 277 for this model (Table 1). The number of dentists suggested to work per district ranges from 14 for the high-density child population to 2 dentists per district. The suggested average number of dentists per district in the 10 highest child population density district was suggested to be 14 dentists.

## 4. Discussion

This study aimed to design a geographic theoretical model for SDS in Al-Madinah, SA, using GIS. This theoretical model was suggested as a solution to tackle the high level of dental caries compounded with the low level of dental care utilization among schoolchildren [5]. Our study showed that the majority of children in Al-Madinah reside in and around the city center with a high population density and where most of the dental health centers are located. The areas shown on the map with the higher number of schools, higher number of students, and dense child population suggest the future location of SDS.

The study proposed two theoretical models that suggest the number of dentists required to operate the SDS per district. With the economic uncertainty that SA is currently facing, a more cost-effective model would be more feasible [23]. The second model is suggested to be more economical with a smaller number of dentists to be employed for SDS. However, a longer waiting time in the first few years of establishing SDs is anticipated. Nevertheless, after the initial period of treatment focus approach on a city level, a transition to a more preventive focus approach is expected after a period of time.

Dental caries is a major public health problem in schoolchildren in SA [4]. Untreated decay in 12–14 schoolchildren in SA found to be significantly impacted daily performances including eating, sleeping, studying, and social contact [24]. These findings are of importance to public health practitioners planners and policy makers. Launching SDS is recommended by this study to tackle dental public health problems in children in Al-Madinah that are predominately high dental caries and low access to dental care [5]. While dental care for all age groups in SA is provided free of charge, the utilization of dental care among schoolchildren is very low [25]. There are many documented barriers to dental care in SA that obstruct the delivery of services to children such as transportation problems, parent availability and waiting time at the dental office [26]. Dental services at public dental clinics provide services to all age groups and are not focused on children. Establishing well-planned SDS could eliminate many of the reported barriers. Furthermore, bringing dental care to schools would increase the access of schoolchildren to healthcare including the most disadvantaged children.

It is important to take into consideration that implementing new health policies in public health, such as the suggested SDS in this study, would face challenges which include but are not limited to: equality, resource allocation, sustainability, population rapid changes, keeping up with research, innovation and technology, and collaboration with other sectors, both public and private [27]. Although dental public health is considered a part of the healthcare system, it does not get the same attention as private medical and dental health. Dental public health economic and health benefits returns are long-term and indirect compared to other health sectors which makes it hard for decision-makers to take it into consideration compared to other shorter-term goals.

Another important challenge to consider before establishing SDS is workforce shortage. One of the recognized and widely used methods applied for assessing workforce outcomes in a given community is dentist-to-population ratio [28]. The dentist-to-population-ratio suggested by the World Health Organization is 1:7500, while the overall city of Al-Madinah reportedly had a higher ratio than the recommended with a dentist-population-ratio of 1:10,848 for primary dental clinics, with a smaller number of dentists to serve the population needs [29,30]. Moreover, the peripheral metropolitan areas of the city had a much higher ratio compared to the central area with 1:44,709 versus 1:7757. This observation is not limited to Al-Madinah, other developing countries are experiencing a similar situation [31]. This workforce shortage is suggested to have a negative impact on the current dental care utilization situation and the future establishment of SDS.

One recommended strategy to solve the shortage of dental staff is to consider employing dental therapists. A shift from dentists to dental therapists has been observed in many developed and developing countries, however, there is a lack of dental therapists in SA [32]. According to the Australian Dental Association in New South Wales, “a dental therapist is a registered primary healthcare professional that provides restorative and preventive dental services to children, adolescents, and teenagers” [33]. Qualified dental therapists were found to increase access to dental care for children under the age of 18 years old [34]. Training dental therapists is less costly and requires less time when compared to training dentists. Employing dental therapists is also more cost-effective. The average annual salary for dental therapists in Australia ranges from AUD 70,000 to 100,000, while the average annual salary for general dentists ranges from AUD 140,000 to 180,000 [35]. It costs approximately 50% less to employ a dental therapist than a dentist. Furthermore, SDS staff is not only limited to dental staff, but it includes other divisions such as administrative staff. With a reported 10.1% unemployment rate in SA in the first quarter of 2022, SDS could be a new employment opportunity for people looking for jobs [16].

The Australian model for SDS provides a guide for the provision of dental care services for school children. Caries prevalence has decreased after founding SDS which is funded by state governments [12]. In the state of Western Australia, the SDS provides free dental care to registered students aged 5 to 16 [36]. The SDS is delivered state-wide through 100 dental therapy centers (DTC), which are co-located with schools that are recognized by the Department of Education. In addition to these fixed sites DTCs, 40 mobile DTCs provide dental care to 150 schools where the accessibility to SDS is limited.

Another example of school-based healthcare is the American model, where school-based health centers are not limited to dental care [7]. Those centers provide a wide range of medical care that includes vision, preventive care, e.g., vaccination, managing healthcare for children with chronic illnesses and coordinating their specialized care, e.g., diabetes and heart disease, and reducing negative health behaviors, e.g., tobacco, use of drugs, and alcohol [7]. Another important aspect of health that those centers provide is mental health. Schools in the United States have become the most common provider of mental health for children [37]. Approximately 70% of school health centers provide mental health services through certified psychologists, clinical social workers, and/or drug abuse counselors [38]. The increased availability of those services has been reported to decrease depression and suicide risk among young adolescents [7,39].

The benefits of the school health centers model in the US extend beyond the healthcare outcomes. Utilization of those services has been associated with positive educational outcomes that include enhanced academic results, e.g., improved GPAs, better attendance, grade promotion, preparation for college, and decreased rates of suspensions from school [40,41]. The relationship between schooling and health has drawn the attention and focus of different organizations and philanthropy work. For example, The Primary School in California in the US is a nonprofit organization that was founded by Priscilla Chan, a pediatrician, an educator, and her husband Mark Zuckerberg, founder of Facebook, now known as Meta. This foundation is based on the belief that education and health systems could work better in cooperating to achieve both goals of hath and educational thriving.

In SA, while dental caries in schoolchildren is highly prevalent, there has been limited national recognition of the problem compounded with little effort regarding its public awareness and prevention [42]. Moreover, there is a lack of effective preventive dental programs for schoolchildren. The present programs focus mainly on oral health education. This approach is insufficient because information alone is not enough in achieving long-term behavioral changes or reducing oral health inequalities [43]. Furthermore, although there are public school health units available, the number of these units is inadequate, and actual contact with children is limited to newly admitted students for school registration purposes only. Increasing access to dental care through SDS would lead to reducing the burden of dental daisies and treatment costs, eventually reducing the government’s budget and preventing future diseases. Caries is a preventable disease, but it still imposes an enormous cost on the Saudi government and requires a large treatment budget. Resource allocation and investing in SDS is a possible solution to such a persistent health problem. A joint effort from The Ministry of Health, Dental schools’ officials, public dental health professionals, the Saudi Dental Society, and private investors to discuss the current caries problem and examine proposed solutions such as the model suggested in this study, carry further extensive database research and create feasible guidelines and plans.

It is worth noting the limitations of SDS. Intern of access, SDS is limited to registered schoolchildren which means that preschool children do not have access to them. In Australia, any treatment that is beyond the scoop of the dental therapist working in SDS is referred to a specialist and the cost would be covered by the family. However, this would not be a major problem in SA because dental care, including specialized dental care, is free of charge. Dental caries and inequalities in oral health still exist among children in Australia to some degree, imposing challenges to the health system.

Some limitations are worth mentioning for this research study. The census data that was obtained from the General Authority of Statistics was the 2010 census data which is considerably old. Therefore, the results presented in this study may not offer the best current evaluation. The proportion of children included in the study was 0–17, which include preschool children in the analysis. Some elements were not considered in the geographic analysis including socioeconomic indicators and economic analysis.

## 5. Conclusions

Dental caries with an alarmingly high prevalence among children in SA is a persistent public health problem. Implementing SDS is recommended to alleviate this problem in Al-Madinah and SA in general. The areas with the highest number of schools, highest number of students, and densest child population as presented in the map suggest the future location of SDS. A guideline for establishing SDS with a framework of the number of dentists needed to hire to meet the child population’s needs was proposed. These results could be used to inform and direct public health policymakers in the utilization of resources to target high-need schoolchildren.

## Figures and Tables

**Table 1:** The number of dentists required for school dental services presented in 2 models for Al-Madinah.

Models	Visits per Year/Child	Total Dental Visits per Year	Service Days	FTE *
Model 1	4.5	1,478,709	98,580	415
Model 2	3	985,806	65,720	277

* Full-time equivalent.

## Data Availability

The data that support the findings of this study are available from The Ministry of Health website.
